# Are Sex Differences in Youth Weightlifting Performance Explained by Fat-Free Mass? A Controlled Analysis

**DOI:** 10.1519/JSC.0000000000005259

**Published:** 2025-10-22

**Authors:** Sylwia Bartkowiak, Sławomir Kozieł, Magdalena Krzykała, Krzysztof Karpowicz, Jan M. Konarski, Bartosz Malak

**Affiliations:** 1 Department of Theory of Sports, Poznan University of Physical Education, Poznan, Poland;; 2 Institute of Sport and Science, Poznan, Poland;; 3 Department of Anthropology, Hirszfeld Institute of Immunology and Experimental Therapy, Polish Academy of Sciences, Wroclaw, Poland;; 4 Department of Methodology of Recreation, Poznan University of Physical Education, Poznan, Poland; and; 5 Department of Neurobiology, Poznan University of Physical Education. Poznan, Poland

**Keywords:** snatch, clean & jerk, squat jump, IMTP, hand grip strength

## Abstract

Bartkowiak, S, Kozieł, S, Krzykała, M, Karpowicz, K, Konarski, JM, and Malak, B. Are sex differences in youth weightlifting performance explained by fat-free mass? A controlled analysis. *J Strength Cond Res* 40(1): 68–75, 2026—This study examined sex differences in weightlifting performance among youth athletes, emphasizing the role of fat‐free mass (FFM), strength, and power. Fifty-three competitive weightlifters (28 male, 25 female) aged 13–15 years were evaluated using bioelectrical impedance analyses to estimate FFM, along with assessments of hand grip strength, squat jump (SJ) power, and isometric mid‐thigh pull force. Competition outcomes for the snatch, clean & jerk (C&J), and total weight lifted were recorded. Male weightlifters demonstrated significantly higher absolute strength outputs compared with female weightlifters (*p* < 0.001). Regression analyses identified SJ power normalized to FFM as the strongest predictor of performance in male weightlifters across the snatch (*r*^2^ = 0.3742, *p* < 0.001), C&J (*r*^2^ = 0.3742, *p* < 0.001), and total (*r*^2^ = 0.3945, *p* < 0.001). In addition, results from the forward stepwise linear regression indicated that waist-to-hip ratio emerged as a significant, sex-specific predictor of C&J performance (*p* > 0.05). These findings underscore the importance of FFM and lower-body explosive strength in youth weightlifting success. However, inherent neuromuscular and biomechanical differences between sexes contribute to persistent performance gaps. The results support the implementation of sex-specific training strategies that target these unique physiologic profiles to optimize performance outcomes in youth weightlifters.

## Introduction

Weightlifting is a renowned Olympic sport, governed by one of the oldest international sports federations, the International Weightlifting Federation (IWF), and its 193 affiliated national associations worldwide (20). Beyond competition, weightlifting and its derivative exercises are central to strength and conditioning programs across various individual and team sports (9,30). Specialization in weightlifting typically begins between 13 and 15 years of age, depending on the sports system and cultural traditions in different countries. In some popular sports such as athletics, swimming, and team sports, early specialization and maturation strongly influence competition outcomes; in contrast, weightlifting adopts a more moderate approach (4,10,21,50). Nonetheless, the relative age effect—a phenomenon where athletes born earlier in the selection period (notably in January) are overrepresented—has been observed among elite weightlifters of both sexes. This effect does not, however, seem to affect success at the Olympic level (25). Interestingly, peak performance is reached at similar ages for both male and female weightlifters (approximately 26.0 years for men and 25.0 years for women), with elite athletes attaining their peak slightly earlier than their subelite counterparts (17).

Body composition is an essential determinant of weightlifting performance, influencing an athlete's ability to generate strength, power, and stability during lifts. In young weightlifters, key anthropometric parameters include fat-free mass (FFM), body mass index (BMI; within the weight category), limb length, and somatotype (5,15,22). A strong correlation observed between lean body mass (a similar parameter to FFM) and performance in the snatch and clean & jerk (C&J) lifts indicates that higher muscle mass contributes to superior outcomes (44). In weightlifting, athletes compete in weight categories, because excessive fat mass (FM) can reduce the strength-to-mass ratio and diminish performance across weight classes (14). Sex differences in weightlifting performance are largely driven by variations in body composition. Boys typically have a higher percentage of lean body mass and greater relative muscle strength, while girls often exhibit a higher proportion of FM, particularly in the lower body, which may aid in balance and stability. Differences in limb proportions and pelvic structure can also necessitate technique adjustments (5,44).

In addition to the influence of body composition and structure on sports performance, the competitive success of an athlete is also determined by a high level of specific physical abilities. Performance assessments such as force in the isometric mid-thigh pull (IMTP), hand grip strength (HGS), power in squat jump (SJ), and countermovement jump have been strongly associated with junior weightlifting success (1,19,47). Junior weightlifters with greater vertical jump power tend to excel in competition, underscoring the importance of power development (6,15). Moreover, foundational strength exercises (including the back squat, front squat, and shoulder press) exhibit robust correlations with weightlifting performance, with elite junior weightlifters consistently outperforming their nonelite counterparts in these exercises (15,31,51).

Although most research has focused on male weightlifters, Galantine et al. (16) reported that FFM in adult athletes accounts for 83% of sex differences observed in the force–velocity profile during sprinting. Similarly, studies indicate that semiballistic exercises (such as the push press, push jerk, and split jerk) yield lower performance values relative to body mass in women than in men (45). In strength exercises, such as shoulder and chest presses, squats, and deadlifts, female upper-body strength is approximately 50–60% of that of male athletes, lower-body strength is 60–70%, and trunk strength is approximately 60% (40).

Given the relatively limited research on youth weightlifters including both sexes, this study aims to compare the anthropometric and fitness characteristics of weightlifters aged 13–15 years, with fine control of maturity status. We hypothesize that differences in FFM are the primary contributors to performance disparities observed between female and male weightlifters.

## Methods

### Experimental Approach to the Problem

This study examined the somatic characteristics and physical performance of youth weightlifters aged 13–15 years, with a particular emphasis on sex differences and the role of FFM. Subjects were recruited from sports clubs to ensure that male and female athletes had comparable training backgrounds and competitive experience. Weightlifting performance was assessed in 2 lifts: snatch and C&J. Independent samples *t*-tests were conducted to evaluate sex differences in continuous variables. Using linear regression, variables showing significant sex differences were then further standardized by FFM, and the resulting standardized residual variances were used in subsequent analyses. This approach aimed to determine whether differences in FFM are the primary contributors to observed performance disparities between male and female youth weightlifters.

### Subjects

The sample consisted of 53 youth weightlifters (25 female and 28 male) aged 13–15 years (mean ± *SD*: 14.5 ± 0.93 years; Table 1). Athletes were recruited from 6 clubs affiliated with the Polish Weightlifting Federation. All subjects had been engaged in a weightlifting training program for at least 1 year, with 3 or more training sessions per week. These athletes were preparing for the Polish Under-15 Championships held in October 2020 and October 2022. Measurements were taken 10 days before each competition. At these championships, the subjects collectively won 19 medals, with a further 24 athletes placing between 4th and 8th, and 10 athletes between 9th and 18th. The sample represented approximately 10% of the total competitors at the championships (*n* = 231 in 2020 and *n* = 280 in 2022). A power analyses (set at *p* = 0.05 and 1-β at 0.80) indicated a required sample size of 58 subjects. Although the final sample was slightly smaller, it consisted of highly trained, completely active, and selectively chosen—members of the Voivodeship Team representing the national-level youth talent pool. This degree of selection minimalized between-subject variability in training exposure and competitive experience. Despite the smaller sample size, several effects were found to be highly significant, indicating relatively strong and practically meaningful relationships between the analyzed variables.

**Table 1:** Descriptive statistics of weightlifters and dependent variables.*†

Variables	Males (*n* = 28)	Females (*n* = 25)	t	Effect size (Cohen's *d*)
	Mean ± *SD*	Mean ± *SD*		
Height	168.30 ± 7.00	162.10 ± 5.40	3.59***	−0.98 (−1.56 to −0.41)
Body mass [kg]	63.50 ± 11.60	64.00 ± 13.50	n.s	0.04 (−0.50 to 0.58)
CA	14.50 ± 1.00	14.50 ± 1.00	n.s	0.00 (−0.54 to 0.54)
PAH [%]	94.20 ± 3.40	98.60 ± 1.80	−5.96***	1.59 (0.97 to 2.21)
Waist-to-hip ratio‡	0.82 ± 0.03	0.75 ± 0.05	6.04***	−1.72 (−2.35 to −1.09)
Hip-to-shoulder ratio‡	2.38 ± 0.17	2.67 ± 0.18	6.11***	1.66 (1.03 to 2.28)
Torso-to-height ratio‡	0.32 ± 0.02	0.31 ± 0.01	3.01**	−0.62 (−1.17 to −0.07)
Torso-to-lower limb ratio‡	0.67 ± 0.05	0.64 ± 0.04	2.51*	−0.66 (−1.21 to 0.11)
Thigh-to-shank ratio‡	0.88 ± 0.07	0.94 ± 0.07	2.45*	0.86 (0.29 to 1.42)
FFM [kg]	51.40 ± 8.00	43.70 ± 6.90	3.71***	−1.03 (−1.60 to 0.45)
HGS [N]	402.40 ± 73.90	306.60 ± 68.20	4.89***	−1.34 (−1.94 to −0.75)
SJ [W]	934.50 ± 192.90	836.20 ± 166.00	n.s	−0.54 (−1.09 to 0.01)
IMTP [N]	1,601.00 ± 538.40	1,002.10 ± 295.60	4.93***	−1.36 (−1.96 to −0.76)
SDS HGS FFM‡	0.31 ± 0.93	−0.34 ± 0.96	2.51*	−0.69 (−1.24 to −0.13)
SDS SJ FFM‡	−0.19 ± 0.99	0.22 ± 0.96	−1.54	0.42 (−0.13 to 0.97)
SDS IMTP FFM‡	0.34 ± 1.09	−0.38 ± 0.71	2.83**	−0.77 (−1.33 to −0.22)
Snatch [kg]	65.20 ± 21.20	44.80 ± 11.00	4.32***	−1.19 (−1.77 to −0.60)
C&J [kg]	80.50 ± 25.80	54.60 ± 12.60	4.56***	−1.25 (−1.84 to −0.66)
Total [kg]	145.80 ± 46.70	99.40 ± 23.40	4.49***	−1.24 (−1.82 to −0.65)

* Sex differences were evaluated using independent samples *t* test with ES calculated using Cohen's *d*.

† CA = calendar age; C&J = clean & jerk; FFM = fat-free mass; HGS = hand grip strength; IMTP = isometric mid-thigh pull; PAH = predicted adult height; SJ = squat jump; SDS HGS FFM = handgrip strength standardized to FFM; SDS SJ FFM = squat jump power standardized to FFM.

‡ Parameters used in regression models; *p < 0.05; **p < 0.01; ***p < 0.001.

All subjects, along with their parents and coaches, were informed about the study details, including potential risks and benefits. Written informed consent was obtained from the parents or legal guardians, and verbal consent was provided by the subjects. The study adhered to the latest version of the Declaration of Helsinki and was approved by the ethics board of Poznan University of Medical Sciences (approval number: 908/16).

### Procedures

All assessments were conducted in a standardized sequence (described below), beginning with antropometric measurments and concluding with the isometric mid-thigh pull test. Data collection occured 10 days prior to competition, during the final training camp. All test were administered on-site under consistent environmenral conditions, with weigthlifters in a non-fatigued state.

#### Anthropometry

Measurements were conducted by a highly trained and experienced anthropologist following the procedures outlined by Martin and Saller (34). Standing height was measured to the nearest 0.1 cm using a stadiometer (GPM, Swiss), and sitting height as the distance from the vertex to the sitting surface. Body mass (BM) was measured, and segmental body composition estimated using bioelectrical impedance analyses (BIA; Tanita MC-780 MA, Japan). The following parameters were also assessed: total body water (kg), FM( kg), FFM (kg·m^−2^), predicted muscle mass (kg), predicted arm muscle mass (kg), and leg muscle mass (kg) (26). All subjects were instructed to refrain from eating or drinking, to empty their bladders, to wear only underwear, and to remove any metal objects before assessment (11). During the BIA measurement, subjects stood upright with their bare feet on the contact electrodes while holding the device's hand-held electrodes. All assessments were conducted in a single morning session.

Given the limited availability of anthropometric data for youth weightlifters, a comprehensive assessment was performed. Specific segment lengths were measured using an anthropometer, including upper limb (acromiale-dactylion), arm (acromiale-radiale), forearm (radiale-stylion), hand (midstylion-dactylion), lower limb (symphysion height), thigh (symphysion-tibiale), calf (tibiale-malleolare internus), and trunk (suprasternale-symphysion).

Breadths were measured using a sliding caliper, including shoulder (biacromial), chest (between the thoracolateral points at the level of the xiphoid process), and hip (bi-iliac breadth). Circumferences were measured with anthropometric tape (to 0.1 cm), including relaxed arm (at the mid-acromiale-radiale level), thigh (distal to the gluteal fold), calf (at the maximal circumference of the medial calf, measured perpendicularly to the leg's long axis), waist (at the narrowest part of the torso), and hip (at the widest part of the gluteus maximus with the subject standing erect). Skinfold thickness was measured using a Harpenden caliper (0.1 mm) at 3 sites: triceps, subscapular, and suprailiac.

Several proportional indices were calculated: relative upper limb length [(upper limb length/body height) × 100], relative lower limb length [(lower limb length/body height) × 100], and the interlimb index [(upper limb length/lower limb length) × 100], and the waist-to-hip ratio (WHR).

### Maturity Status

Biologic age prediction was used to differentiate between subjects who were chronologically similar but biologically distinct. Prediction equations were based on the proposal by Khamis and Roche (23), incorporating the chronological age, height, and weight of each subject, and the mid-parent height of their biologic parents. Parents' height was recorded from a personal identification survey taken during a brief interview. Reported parental heights were adjusted for the tendency of overestimation using the sex-specific equations of Epstein et al. (13). The current height of each weightlifter was expressed as a percentage of their predicted mature height to provide an estimate of biologic maturity status (23,32). Maturity status indicated that both sex groups predominantly exhibited normal maturation rates. The only exceptions showing early maturation were 2 female athletes with a difference of 4.16 and 2.83 years, respectively, recorded between their current biologic age and calendar age, indicating that they were fully mature, and 1 male athlete who was biologically 1.25 years older than his current age (Table 1). This approach reduced the potential bias introduced by age-related physiologic heterogeneity, strengthening the validity of our conclusions.

### Fitness Tests

After a standardized warm-up, each weightlifter completed a series of physical fitness tests. A single examiner administered each test. The tests were performed in the following order.

#### Hand Grip Strength

Hand grip strength was measured in each hand using a Takei 5401 Digital Dynamometer (Takei Scientific Instruments Co., Ltd., Tokyo, Japan). The test was conducted with the athlete standing, with the arm held by the side (without touching the body), and the elbow fully extended. Three trials were conducted for each hand, with a 1-minute rest between trials to avoid neuromuscular fatigue. Athletes were instructed to apply maximal grip force. The peak strength value for each hand was recorded, and the higher value (i.e., from the stronger hand) was used for analyses (43).

#### Squat Jump

The SJ, an indicator of lower-body power, was measured using a Tendo Weightlifting Analyzer (Tendo Sports Machines, Trencin, Slovak Republic (46,49)). The jump was performed with the athlete's hands on their hips (12,33), with a 1-minute rest between jumps. The average power output across the 3 jumps was used for subsequent analyses.

#### Isometric Mid-Thigh Pull

The IMTP test was used to estimate maximal isometric strength. This test was conducted with a custom-made device equipped with an AXIS FC50K force meter (AXIS, Gdańsk, Poland). Each weightlifter was positioned on a platform beneath a fixed bar. Knee and hip joint angles were set at approximately 125–145° and 140–150°, respectively, based on each athlete's optimal power position during the clean phase of the lift. Weightlifters completed warm-up trials at 50 and 75% of their perceived maximum strength, with a 1-minute rest between trials. A 3-minute rest was provided before each of the 2 maximal pull efforts, which lasted 3–5 seconds (35,48). Athletes were familiar with the test protocol and were instructed to pull as fast and as hard as possible. Peak isometric force was recorded for analyses (1).

#### Competition Results

The final results from the Polish Under-15 Championships, including the snatch, C&J, and the combined total, were obtained from the official website of the Polish Weightlifting Association (42) and included in the analyses.

### Statistical Analyses

All variables are reported as sex-specific means ± *SD*s. The normality of each measurement was tested using the Shapiro–Wilk test. Intraclass correlation coefficients (ICCs) were calculated for grip strength in the dominant (ICC = 0.95–0.99) and nondominant (ICC = 0.95–0.98) hands, as well as for mean power during the SJ (ICC = 0.97–0.99). The maximal isometric strength measured was used in subsequent analyses (1).

Independent sample *t*-tests were used to assess sex differences for continuous variables. The parameters that exhibited high sex differences were standardized to FFM (derived from BIA) using linear regression; the standardized residual variances were used in further analyses.

Given the high intercorrelations among independent variables and the relatively small sample size, forward stepwise regression was performed using 3 models. These models were implemented within a generalized linear model framework, with the snatch, C&J, and total serving as dependent variables in 3 separate analyses. A logit function was used as the link function.

In the first model, independent variables included sex, SJ, HGS, and maximal isometric strength standardized to FFM, WHR, and 4 body proportion ratios: hip-to-shoulder width, torso-to-lower limb length, torso length-to-height, and thigh-to-shank length. In addition, second-order interaction effects between all independent variables and sex were included.

An inclusion criterion of *p* = 0.05 was used for entering an independent variable or second-order interaction in the stepwise regression. Significant second-order interaction effects are presented in graphical form. The effect size, measured using Cohen's *d* (d henceforth), was used to compare differences in anthropometric and fitness parameters between male and female athletes with different sample sizes. All calculations were performed using STATISTICA 13.0 (TIBCO Software Inc. (2017); http://statistica.io.) and RStudio 4.0.2 (RStudio, PBC; Boston, MA).

## Results

Descriptive statistics for the examined variables are presented in Table 1. Most variables showed significant sex differences, except for age, BMI, and SJ adjusted for FFM.

All competition and fitness test results were correlated with FFM in both sexes (*R*^2^ = 0.34–0.73 for male athletes and 0.16–0.64 for female athletes, Tables 2 and 3).

**Table 2:** Pearson correlations between competition and fitness variables and FFM in male athletes.*

Variables	*r*	*R* ^2^	*t*	*p*
HGS [N]	0.71	**0.50**	5.11	<0.001
SJ [W]	0.85	**0.72**	8.18	<0.001
IMTP [N]	0.54	**0.29**	3.27	0.003
Snatch [kg]	0.61	**0.37**	3.88	<0.001
C&J [kg]	0.58	**0.34**	3.63	0.001
Total [kg]	0.60	**0.36**	3.79	<0.001

* C&J = clean & jerk; IMTP = isometric mid-thigh pull; HGS = hand grip strength; SJ = squat jump; FFM = fat-free mass.

**Table 3:** Pearson correlations between competition and fitness variables and FFM in female athletes.*

Variables	*r*	*R* ^2^	*t*	*p*
HGS [N]	0.62	**0.38**	3.79	<0.001
SJ [W]	0.80	**0.64**	6.39	<0.001
IMTP [N]	0.40	**0.16**	2.09	0.048
Snatch [kg]	0.57	**0.33**	3.33	0.003
C&J [kg]	0.61	**0.37**	3.65	0.001
Total [kg]	0.60	**0.35**	3.56	0.002

* C&J = clean & jerk; IMTP = isometric mid-thigh pull; HGS = hand grip strength; SJ = squat jump; FFM = fat-free mass.

**Figure 1.**
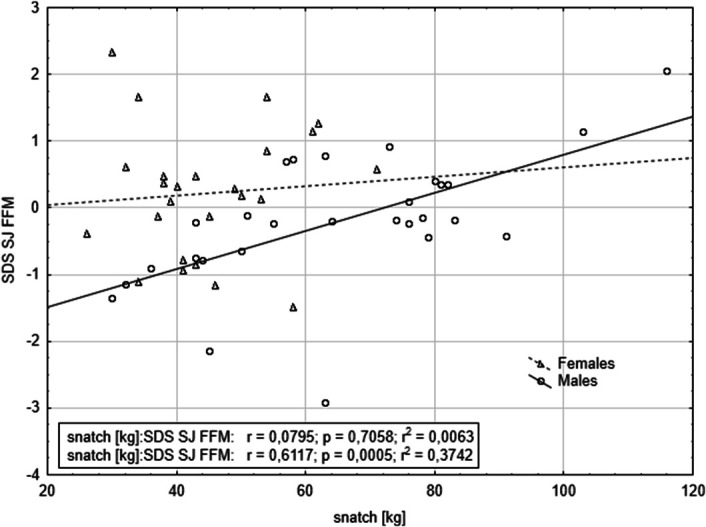
The relationship between squat jump power adjusted for fat-free mass and snatch performance, stratified by sex.

**Figure 2.**
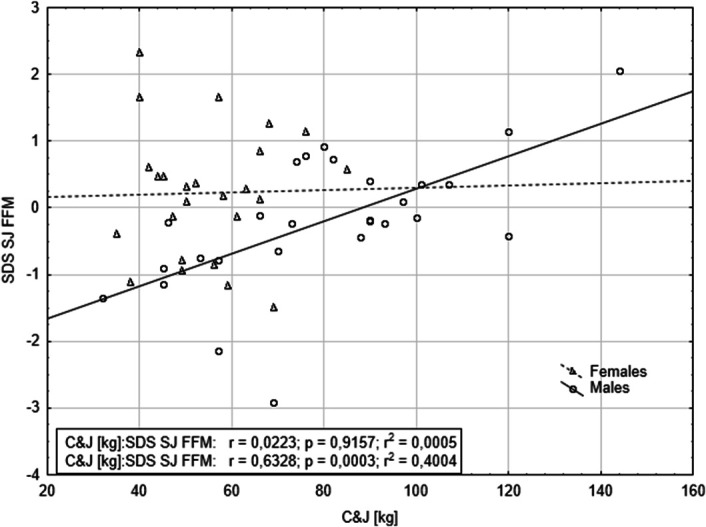
The relationship between squat jump power adjusted for fat-free mass and clean and jerk performance, stratified by sex.

**Figure 3.**
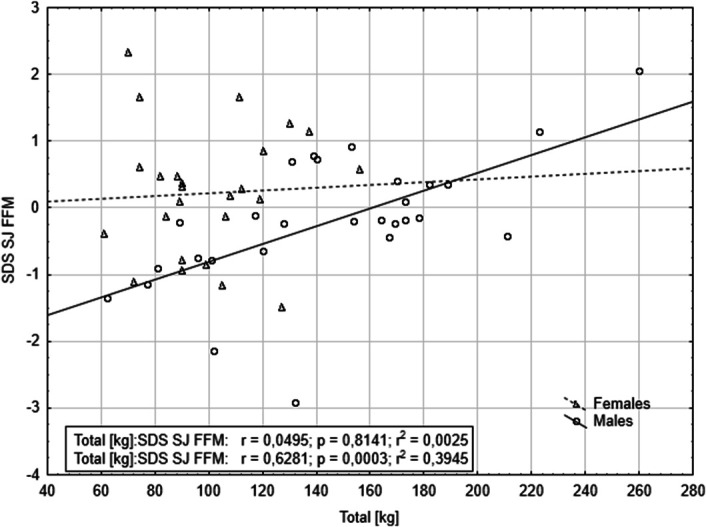
The relationship between squat jump power adjusted for fat-free mass and total performance, stratified by sex.

Table 4 presents the results of forward stepwise regressions, which included all variables and selected second-order interactions from the first model. The analyses revealed significant interactions between SJ adjusted for FMM and sex, affecting snatch performance. Similarly, SJ adjusted for FFM and WHR interacted with sex, influencing C&J performance (Table 5). Finally, only 1 interaction—SJ adjusted for FFM with sex—significantly affected total weightlifting performance (Table 6). These results indicate that SJ adjusted for FFM is a significant predictor of snatch, C&J, and total weightlifting performance, with sex-dependent effects (Figures 1–3).

**Table 4:** Forward stepwise linear regression results for snatch (kg) as the dependent variable.*†

Variables	χ^2^ Wald's (df)	*p*
Sex*TL/LL	4.48 (2)	0.1066
Sex*SDS SJ FFM	21.45 (2)	<0.0001
Sex*SDS HGS FFM	4.18 (2)	0.1236
Sex*WHR	3.36 (2)	0.1860

* Only significant effects are shown.

† TL/LL = torso-to-lower limb ratio; SDS SJ FFM = squat jump power standardized to FFM; SDS HGS FFM = handgrip strength standardized to FFM; WHR = waist-to-hip ratio.

**Table 5:** Forward stepwise linear regression results for C&J (kg) as the dependent variable.*†

Variables	χ^2^ Wald's (df)	*p*
Sex*SDS HGS FFM	2.38 (2)	0,3041
Sex*SDS SJ FFM	25.55 (2)	<0,0001
Sex*WHR	6.40 (2)	0,0407
Sex*HSWR	2.17 (2)	0,3377

* Only significant effects are shown.

† HSWR = hip-to-shoulder width ratio; SDS HGS FFM = handgrip strength standardized to FFM; SDS SJ FFM = squat jump power standardized to FFM; WHR = waist-to-hip ratio.

**Table 6:** Forward stepwise linear regression results for total (kg) as the dependent variable.*†

Variables	χ^2^ Wald's (df)	*p*
WHR	1.20 (2)	0.2726
Sex*SDS SJ FFM	30.74 (2)	<0.0001
Sex*TCR	0.83 (2)	0.6590
Sex*THR	4.04 (2)	0.1325

* Only significant effects are shown.

† SDS SJ FFM = squat jump power standardized on FFM; TCR = thigh-to-calf ratio; THR = torso-to-height ratio; WHR = waist-to-hip ratio.

## Discussion

This study examined the anthropometric and fitness characteristics of youth weightlifters aged 13–15 years, with a particular focus on sex differences and the predictive role of FFM in determining performance. Our findings indicate that FFM is a key predictor of performance, as evidenced by significant correlations between FFM and both competition results and fitness test outcomes. Although male athletes exhibited superior absolute strength and power—as measured by the snatch, C&J, and total weight lifted—adjusting for FFM reduced, but did not eliminate, these sex differences. This suggests that, in addition to FFM, other factors contribute to the observed performance disparities, which may include neuromuscular activation, muscle architecture, and hormonal influences (18).

Galantine et al. (16) reported that FFM explained 83% of the variance of the sex differences in the force–velocity profile during sprinting. The disparity between our results may be attributed to the distinct nature of resistance encountered in sprinting versus weightlifting; sprinters move only their BM, whereas weightlifters move an external, relatively heavy load. Nevertheless, a strong correlation between FFM and SJ performance was also observed, reinforcing the role of lower-body power in explosive movements. In addition, Ben Mansour et al. (3) reported that body fat in young adults accounts for 30–70% of the observed differences in performance and power outcomes between sexes during jump tests. This suggests that, beyond muscle mass, differences in body composition—specifically fat distribution—may significantly contribute to sex-based variations in explosive power.

Among adolescent school athletes (16.8 ± 0.8 years old)—primarily trained at handball, soccer, and volleyball clubs but without experience in strength training (Tanner stages 4–5)—an 8-week strength training program resulted in greater improvements in maximal isometric force (N) and rate of force development (N × m·s^−1^) of the leg extensors in female athletes compared with male athletes. This sex difference was also maintained when expressed relative to BM. These findings suggest that the trainability of strength in adolescent girls may be more pronounced and effective compared with boys (38). However, responses to maximal leg strength training seem to be similar across a wide age range, from 20 to >70 years old (24). In contrast, Collins et al. (8) reported that boys exhibited greater gains in muscle power after resistance training compared with girls. However, this finding is not consistently supported across the literature, because only 1 meta-analysis has demonstrated significant sex differences in strength training effects (2,27,28).

The extensive literature reviewed by Nuzzo (40) underscores that differences in muscle mass and fiber type and composition are fundamental drivers of sex disparities in strength and power among adults. Adult female athletes typically possess 65–80% of the lean body mass, 68–75% of the FFM, and 60–65% of the skeletal muscle mass compared with male athletes. In addition, their upper limb lean mass ranges from 55 to 65%, and lower limb lean mass from 62 to 75%, of that of their male counterparts, consistent with findings from field strength tests (40). Delving deeper into biologic conditions, male athletes exhibit greater cross-sectional areas for all fiber types, higher distribution percentages of fast-twitch fibers, and elevated type II/I and IIA/I fiber ratios. Conversely, female athletes demonstrate greater area and distribution percentages of slow-twitch fibers, along with a higher type I/II ratio (41).

Animal studies indicate that these distinctions extend to muscle fibers and motor units. Specifically, male rodents exhibit 11–42% more muscle fibers and 7–29% larger fiber diameters in hindlimb muscles, such as the tibialis anterior, flexor digitorum brevis, and soleus compared with female rodents. The most pronounced differences are observed in the tibialis anterior, a typical fast-twitch muscle, whereas the smallest differences are found in the soleus, a typical slow-twitch muscle (36). For instance, in the medial gastrocnemius muscle, the number of muscle fibers innervated by a single motoneuron is approximately 26% higher in male rodents than in female rodents. Consequently, disparities in muscle fiber morphometric parameters and motor unit innervation ratios contribute to the enhanced force production observed in individual motor units (37). Moreover, fast-twitch fatigable and fast-twitch resistance motor units exhibit shorter contraction and half-relaxation times in male rodents compared with female rodents, whereas slow-twitch motor units show no significant differences in these parameters (7). Adaptations in the contractile properties of different motor unit types may vary after strength training (29). These physiologic differences may underlie the greater maximal force production and rate of force development observed in male athletes, further explaining performance disparities between sexes in weightlifting and other explosive strength-based sports.

Instead of relying on routine anthropometric parameters, which typically increase with height and body mass, we focused on body proportionality. Certain leverages may confer biomechanical advantages and correlate with competition performance in weightlifters (39). Although male and female athletes differed in all examined ratios, only the WHR significantly influenced C&J performance, with distinct differences between sexes. This finding suggests that body proportions and mass distribution, particularly in the waist and hip regions, affect C&J performance in a sex-dependent manner.

In summary, FFM is a major predictor of weightlifting performance in youth athletes, as demonstrated by its significant correlations with snatch, C&J, total weight, and SJ performance. Although male athletes exhibited higher absolute strength, adjustments for FFM revealed persistent sex differences, highlighting the contributions of neuromuscular and morphologic factors such as muscle mass distribution in the waist and hip regions. These findings emphasize the importance of FFM and lower-body power in youth weightlifting and suggest that sex-specific training strategies may further optimize performance.

The relatively small sample size and the fact that all subjects were from a single country may limit the generalizability of our findings to broader populations. Furthermore, the study offers only a cross-sectional snapshot, limiting insights into how training influences maturity-related performance changes over time. A longitudinal approach is needed to better capture growth-related adaptations in both male and female athletes. In addition, the use of BIA is a limitation, because it is less accurate than methods such as dual-energy X-ray absorptiometry (DEXA) or MRI for estimating body composition. Future research should, therefore, prioritize increasing the sample size and incorporating athletes from diverse countries to enhance the generalizability of findings. In addition, longitudinal tracking of performance changes throughout growth and maturation is crucial for understanding sex-specific developmental trajectories. To draw more definitive conclusions, biomechanical analyses of lifting techniques should be conducted to elucidate the differential effects of SJ power in male and female athletes. Furthermore, more precise body composition assessment techniques, such as DEXA, are recommended to validate BIA findings. These methods would enable more accurate evaluations of muscle mass volume involved in maximal force production during joint-specific movements. Such analyses could offer deeper insights into how muscle mass influences force production in male and female athletes. In addition, surface electromyography data could provide valuable information on the neuromuscular properties underlying force production, particularly in relation to sex differences. Further studies should also explore how power-based training interventions differentially affect male and female youth weightlifters.

**Practical Applications**

These findings have direct implications for the training and coaching of youth weightlifters. In particular, lower-body power—quantified as SJ performance adjusted to FFM—emerged as a key predictor of performance. This parameter can be assessed concurrently with 1 repetition maximum tests for the snatch and C&J, thereby providing an objective marker of physical improvement independent of technique, which is often less stable during the early stages of training. Consequently, coaches should consider incorporating routine assessments of SJ power adjusted to FFM into performance monitoring protocols, given its reliability as an indicator of explosive strength potential. Moreover, the observed sex-dependent differences underscore the need to tailor training programs to the distinct developmental and biomechanical characteristics of young male and female athletes. For instance, interventions specifically designed to enhance lower-body power may be particularly beneficial for male athletes, whereas training programs for female athletes might emphasize the optimization of technique and body composition to maximize force transfer during lifts.
